# Septic Arthritis of the Acromioclavicular Joint: A Case Report

**DOI:** 10.21980/J8VP9N

**Published:** 2024-01-31

**Authors:** Serena Tally, Michael Head, Kerri Kraft

**Affiliations:** * University of California, Irvine, Department of Emergency Medicine, Orange, CA

## Abstract

Septic arthritis of native joints is uncommon, but the condition can be threatening to life and limb if left untreated.^1^ Septic arthritis of the acromioclavicular (AC) joint of the shoulder is particularly rare and has only appeared sparsely in medical literature, mainly through individual case reports. Early recognition and treatment of the condition is vital, but diagnosis of septic AC arthritis can be difficult due to its presentation with vague symptoms and nonspecific laboratory findings. This case report describes the care of a patient with poorly managed diabetes who presented to the emergency department with one month of pain and swelling of the left shoulder and two weeks of pain and swelling in the right ankle. Imaging revealed fluid in the AC joint, and laboratory evaluation showed an elevation in inflammatory markers, including leukocyte count, C-reactive protein (CRP), and erythrocyte sedimentation rate (ESR). The patient’s hospital course was complicated by methicillin-sensitive Staphylococcus Aureus bacteremia without evidence of sepsis. The patient underwent open debridement and washout of both the ankle and AC joint without complication. After recovery, the patient was discharged to a rehabilitative center with IV antibiotics and weekly follow up care with infectious disease specialists. This case illustrates the importance of early diagnosis and treatment of septic arthritis, even in less common joint spaces, to prevent progression of this dangerous disease.

**Topics:**

Septic arthritis, acromioclavicular joint, diabetes, bacteremia.

[Fig f1-jetem-9-1-v9] [Fig f2-jetem-9-1-v9] [Fig f3-jetem-9-1-v9] [Fig f4-jetem-9-1-v9] [Fig f5-jetem-9-1-v9]

**Figure f1-jetem-9-1-v9:**
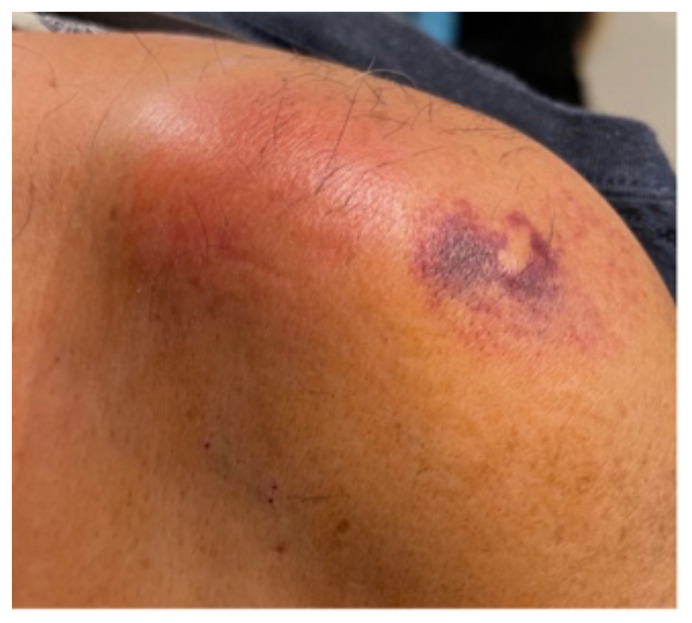


**Figure f2-jetem-9-1-v9:**
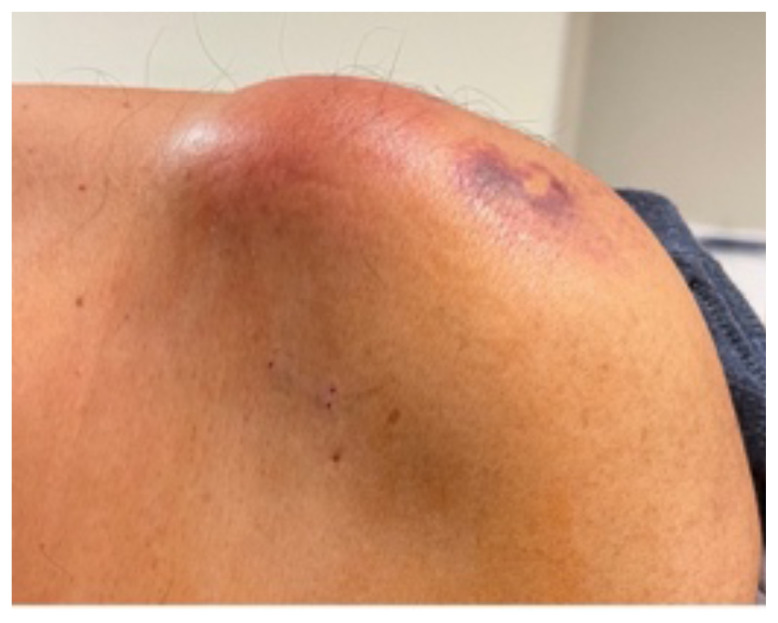


**Figure f3-jetem-9-1-v9:**
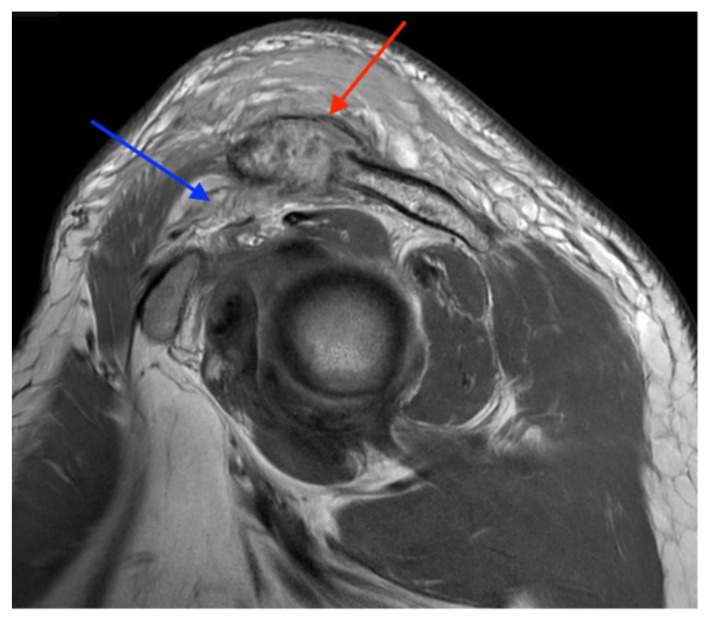


**Figure f4-jetem-9-1-v9:**
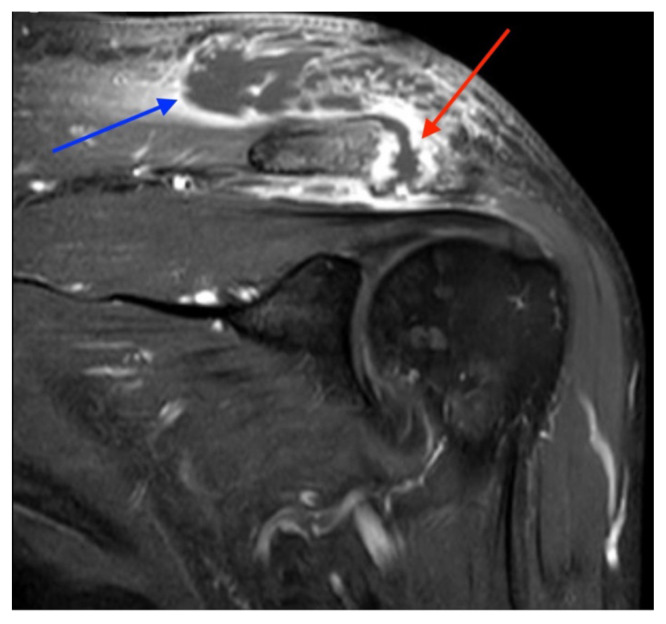


**Figure f5-jetem-9-1-v9:**
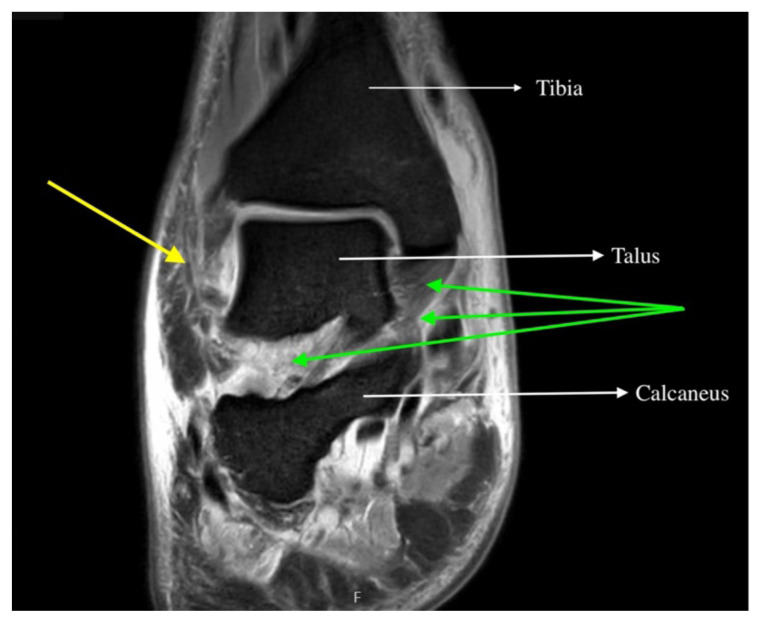


## Brief introduction

Septic arthritis (SA) is the inflammation of a joint due to a bacterial infection. Occurring more often in prosthetic joints, septic arthritis of native joints is rare, with approximately two cases per 100,000 people occurring each year.^1^ Septic arthritis of the acromioclavicular (AC) joint of the shoulder is a particularly rare condition that has appeared in only a limited number of case reports: a 2018 review found just 28 reported cases, but the exact number of cases is likely larger.^2^ Of all septic arthritis cases, only a few reflect infection of the AC joint specifically, meaning that this condition has rarely been documented in the literature and can be more difficult to diagnose. In comparison, septic arthritis of the larger and nearby glenohumeral (GH) joint is more common, comprising twelve percent of all monoarticular native septic joints found in a 2020 retrospective study that is the largest systematic review of native joint SA to date.^3^ This condition typically occurs in immunocompromised patients.^4^,^5^ One systematic review found that over 75% of patients diagnosed with septic arthritis were immunocompromised due to diabetes, cancer, chemotherapy, or intravenous (IV) drug use.^2^ Because this condition rarely presents in young, healthy individuals, diagnosis in this demographic may be more challenging. However, with increasing rates of significant medical comorbidities such as diabetes and IV drug use in the general public, the likelihood that Emergency Department (ED) physicians will encounter patients with septic arthritis is also increasing.^6^,^7^ Timely recognition and management of this condition is crucial since joint destruction, systemic infection, and even death may result if left untreated.

Septic arthritis often presents with fever, erythema, edema, and tenderness over the affected joint.^2^,^4^ However, symptoms may vary and the patient we present here experienced only minor pain and swelling. Both active and passive shoulder movement may be limited due to pain.^8^ This condition can be difficult to diagnose because the clinical picture may be mistaken for degenerative arthritis or pathology of the glenohumeral joint.^3^ Physical exam can help differentiate between pathology of the glenohumeral joint by determining which specific movements illicit pain. The AC joint is typically more affected by external rotation and flexion at the shoulder above 90 degrees, while the glenohumeral joint is affected by any movement at the shoulder, including flexion, extension, abduction, adduction, external rotation, and internal rotation. Diagnosis is made by clinical features along with laboratory values, radiological findings, and culture analysis of joint aspirates. Patients may present with increased inflammatory markers, including an elevated leukocyte count, CRP, and/or ESR. X-ray imaging may show an enlarged joint space and/or destruction of the joint.^9^ Ultrasound and magnetic resonance imaging (MRI) may both show effusion and/or capsular distention of the joint.^10^ However, MRI has been shown to be more specific than ultrasound and X-ray in detecting abscesses and effusions in smaller joints, such as the AC joint.^11^ While a presumptive diagnosis may be made based on these clinical, laboratory, and radiological findings, the gold standard for diagnosis of a septic AC joint is arthrocentesis and bacterial culture of the joint aspirate.^1^

Definitive diagnosis from bacterial cultures takes significant time to result and subsequently may delay treatment. Therefore, it is important to begin empiric treatment with a broad-spectrum antibiotic that covers the most common bacterial infections while culture results are pending. *Staphylococcus aureus* is the pathogen most often isolated from septic AC joint aspirates, followed by *Streptococcus pneumoniae*.^2^,^4^,^9^ Other identified pathogens have included *Staphylococcus epidermidis, Streptococcus viridans*, and *Group B streptococcus.*^2^ Patients are typically started on broad spectrum antibiotics with MRSA coverage, such as IV Vancomycin and IV Piperacillin/Tazobactam, then deescalated as cultures result.^12^ Current guidelines also recommend surgical wound washout and debridement on a case-by-case basis, necessitating urgent orthopedic surgery consult as well.^2^,^5^

## Presenting concerns and clinical findings

The patient is a 58-year-old male who presented to the emergency department (ED) with one month of sharp left shoulder pain, swelling and redness. He first noticed the pain in his left bicep while driving, and it initially felt like a “dull soreness.” Over the next month, the pain progressively worsened, and he noticed swelling and redness localizing around the superior aspect of the left shoulder. The pain was constant and home interventions – which included ibuprofen, naproxen, and cupping – provided no relief. He denied any preceding trauma or other inciting events. He had seen an outside physician for the pain and was told that he had “fluid in the joint,” and to come to the ED to get the fluid removed. The patient also reported two weeks of atraumatic right ankle pain. He denied numbness or tingling in any extremity. He denied fever, chills, or other systemic symptoms at that time. The patient’s medical history was significant for poorly controlled diabetes mellitus with a recent glycosylated hemoglobin (HbA1C) of 12. He denied a history of IV drug use or previous joint pathology such as septic arthritis, osteoarthritis, or rheumatoid arthritis.

## Significant findings

The patient had significant pain in the left shoulder upon examination. Additionally, there was edema and erythema of the skin over the AC joint, and ecchymosis over the lateral shoulder that was characteristic of skin findings from cupping (as shown in photographs). Pain with range of motion began once the patient reached 90 degrees of shoulder abduction and flexion. He also had significant pain with external rotation of the shoulder. He was tender to palpation over the AC joint, but nowhere else in the left upper extremity or trunk. Neurological examination showed no decrease in sensation or strength in the left upper extremity. Additional physical exam findings were unremarkable. The patient’s temperature was 98.4 degrees Fahrenheit, pulse was 90 beats per minute, blood pressure was 105/64 millimeters of mercury. Lab work in the ED showed a leukocyte count of 16.8, CRP of 27, ESR of 111, and lactate of 1.0.

Examination of the right ankle also showed erythema, edema, and decreased range of motion due to pain. He had mildly decreased sensation throughout the foot, with otherwise intact sensation to light touch in the dermatomal regions corresponding to the second lumbar to the first sacral spinal levels. Dorsalis pedis (DP) and posterior tibial (PT) pulses were normal. No crepitus, bullae or open wounds were found in the area.

Magnetic resonance imaging (MRI) with contrast was obtained of the shoulder and ankle, and results from both scans showed findings consistent with septic arthritis complicated by intraarticular abscesses. The MRI of the patient’s left acromioclavicular joint is shown as both a T1-weighted sequence in sagittal view and T2-weighted sequence in coronal view. The images show effusion (the dark fluid denoted by the red arrow) with an adjacent fluid collection (blue arrow). A T2-weighted MRI in coronal view of the patient’s right ankle showing multiple effusions (green arrows) and a fluid collection along the medial tibial cortex and subcutaneous tissues (yellow arrow). Blood cultures obtained in the ED grew *methicillin-*susceptible *Staphylococcus aureus (*MSSA). Transthoracic echocardiogram (TTE) was performed, and results indicated no sign of endocarditis or valvular vegetations.

## Patient course

Based on the history, physical examination and lab findings, a preliminary diagnosis of septic arthritis of both the shoulder and ankle was made. The differential diagnoses included septic arthritis, osteoarthritis, rheumatoid arthritis, cellulitis, bursitis, fracture, or dislocation. Providers in the ED attempted aspiration of the right ankle and left shoulder at bedside without success. Orthopedic surgery was consulted, and they believed the patient’s findings likely represented septic arthritis of AC joint. They proposed the right ankle findings were from hematogenous seeding of the bacteria, causing cellulitis of the right ankle. They recommended against treating with IV antibiotics in the ED given that the patient showed no signs of systemic infection, and they preferred to attempt their own arthrocentesis prior to antibiotics to obtain reliable culture results. The patient was admitted to the hospital that night for continued monitoring. On hospital day two, the patient was started on broad spectrum antibiotics for presumed septic arthritis without plans to go to the operating room. An MRI was ordered on admission for further evaluation of the joint, but results were not available until hospital day four.

Orthopedic surgeons performed an open washout and debridement procedure of the right ankle and left shoulder in the operating room (OR) under general anesthesia once MRI results were obtained and demonstrated evidence of intraarticular abscesses in addition to the presumed septic arthritis. The right ankle and left AC joint were successfully debrided, irrigated, and packed with one gram of vancomycin powder. The patient was started on cefazolin through a peripherally inserted central catheter (PICC) for six weeks to target the MSSA bacteremia. He was discharged with this treatment regimen and was subsequently followed weekly by the infectious disease team.

On follow up, the patient reported significant improvement in his symptoms. His left shoulder was back to baseline with no residual pain or loss of function. The right ankle had a small, approximately one centimeter wound that was still healing, but the patient reported he was otherwise back to baseline. He was followed by infectious disease physicians in clinic for six weeks, at which point his course of antibiotics was completed and inflammatory markers had trended downward. He also followed up in primary care clinic for closer management of his underlying diabetes.

## Discussion

We present here a case of septic arthritis of the AC joint in a 58-year-old male. Septic arthritis of the AC joint is a rare condition that has only been described in a limited number of case reports to date. Given the patient’s poorly controlled diabetes illustrated by his HbA1C of 12, he falls in the typical patient demographic this condition appears in: individuals who are immunocompromised due to either chronic disease, immunosuppressants, chemotherapy, or IV drug use. However, it is important for providers to be aware that septic arthritis can also occur in patients who are younger and have no other comorbidities. This is especially relevant given the increasing number of individuals in the US who use IV drugs, and those who lack access to consistent primary care and may have undiagnosed comorbid diseases, such as cancer, atherosclerosis, or diabetes. This condition may be rare, but it can cause significant morbidity and mortality if left untreated.

It may be difficult to diagnose this condition given its rarity and often vague presenting symptoms such as general shoulder pain and nonspecific lab markers of inflammation. Additionally, it may be difficult to differentiate septic arthritis of the AC joint from that of the GH joint, which is a slightly more commonly seen infection. MRI imaging helped localize this patient’s infection by showing effusion of the AC joint specifically. If imaging is not readily available, performing a cross-body adduction test has been shown to be highly sensitive and specific to identify pathology of the AC joint over other joints in the shoulder.^13^ This physical exam maneuver involves having the patient flex and adduct the affected arm across their body such that the patient’s bicep muscles meet their chest. If this maneuver elicits pain in a patient with additional signs of infection (such as fever, elevated inflammatory lab markers, or point tenderness directly over the joint), a diagnosis of AC joint septic arthritis should be high on the differential.

To arrive at this diagnosis, we took into consideration our patient’s immunocompromised state, history of no trauma to the shoulder, clinical findings of erythema and pain over the superolateral aspect of the shoulder, laboratory testing that showed increased inflammatory markers, and imaging showing effusion of the AC joint. The gold standard for diagnosis of septic arthritis is arthrocentesis to obtain bacterial cultures either at bedside or in the OR. Empiric treatment with a broad-spectrum antibiotic that covers the most common bacteria is recommended while awaiting definitive culture results. *Staphylococcus aureus* is the pathogen most often isolated from septic AC joint aspirates, followed by *Streptococcus pneumoniae*. The patient agreed to undergo washout and debridement of his shoulder and ankle to prevent further disease progression. Surgical intervention such as this may not always be necessary, and doing so should be decided jointly by the patient and providers on a case-by-case basis. In this case, the decision to go to the operating room was made based on the patient’s lack of clinical improvement at hospital day 4 and the presence of likely abscesses noted on MRI requiring operative drainage.

Risk factors for septic arthritis of the AC joint are the same as those for all native joints. They include immunosuppression (due to chronic disease, IV drug use, or use of immunosuppressive medications) and/or prior disease of the affected joint (such as rheumatoid arthritis, osteoarthritis, gout and/or recent trauma). The likely pathogenesis of septic arthritis in individuals who are immunocompromised or who use IV drugs is hematogenous spread of the organism from elsewhere in the body. Because of this hematogenous spread, it is important to obtain transthoracic or transesophageal echocardiography to rule out coexisting cardiac vegetations or infective endocarditis. This is an uncommon yet important cause of septic arthritis, since infective endocarditis can cause heart failure, stroke, pulmonary embolism, abscesses and more.^14^ One of the other key takeaways from this case is the importance of obtaining blood cultures on any patient who presents to the ED with symptoms of septic arthritis and no history of prior joint disease. This patient was afebrile and did not appear to have a systemic infection, but he was found to have MSSA bacteremia without sepsis.

Another interesting aspect to our case is the presence of concomitant infection of both the right ankle and left AC joint. This may have resulted from hematogenous spread from the patient’s original infection of the AC joint or both infections may have seeded from another infectious source within the body. While we may not be able to determine how these simultaneous infections began, the presence of an additional site of infection in this patient does demonstrate the potential of untreated septic arthritis to seed abscesses elsewhere in the body. Overall, septic arthritis of the AC joint is a rare but potentially fatal condition if allowed to progress without intervention. Therefore, it is important to document reports of AC joint septic arthritis to illustrate for future providers the variety of ways this condition may present in the ED.

## Supplementary Information
















