# Supplementary figures and images for: Septic Arthritis of the Acromioclavicular Joint: A Case Report
Source: J Educ Teach Emerg Med. 2024 Jan 31;9(1):V9–V14. doi: 10.21980/J8VP9N (PMC10854883; doi:10.21980/J8VP9N)

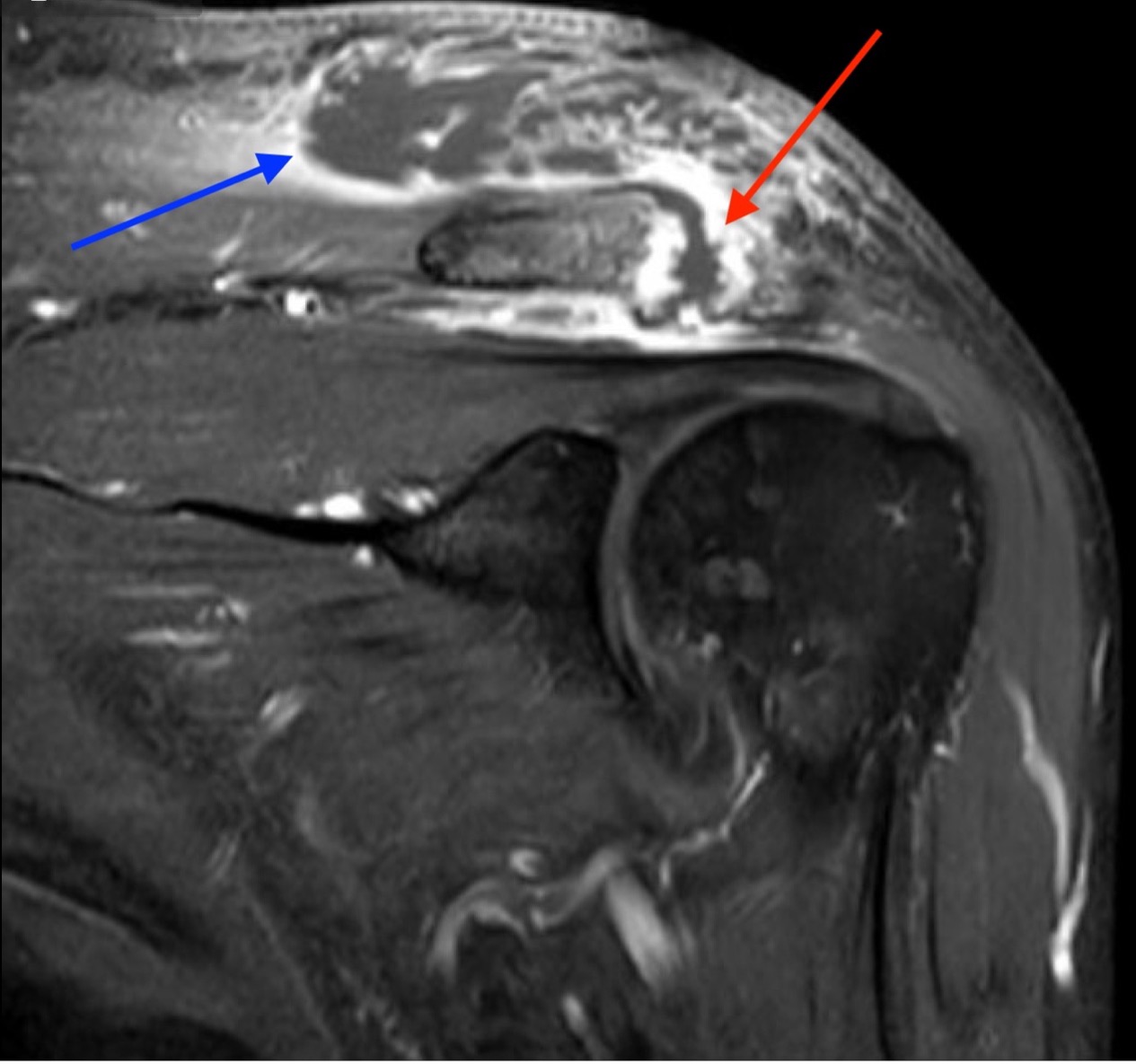

Supplement: Supplementary file 1 [file jetem-9-1-V9-supp1.jpg]

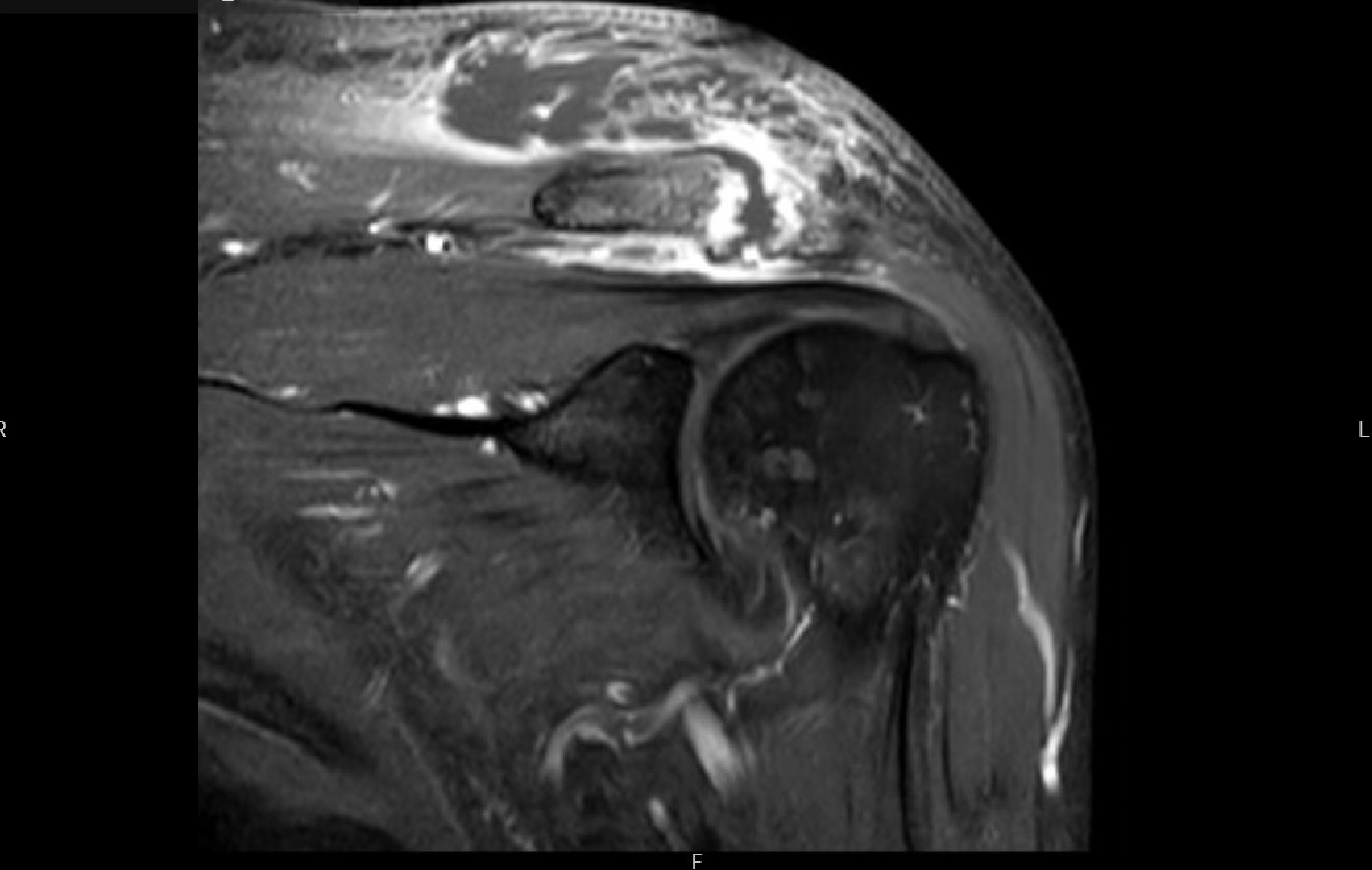

Supplement: Supplementary file 2 [file jetem-9-1-V9-supp2.jpg]

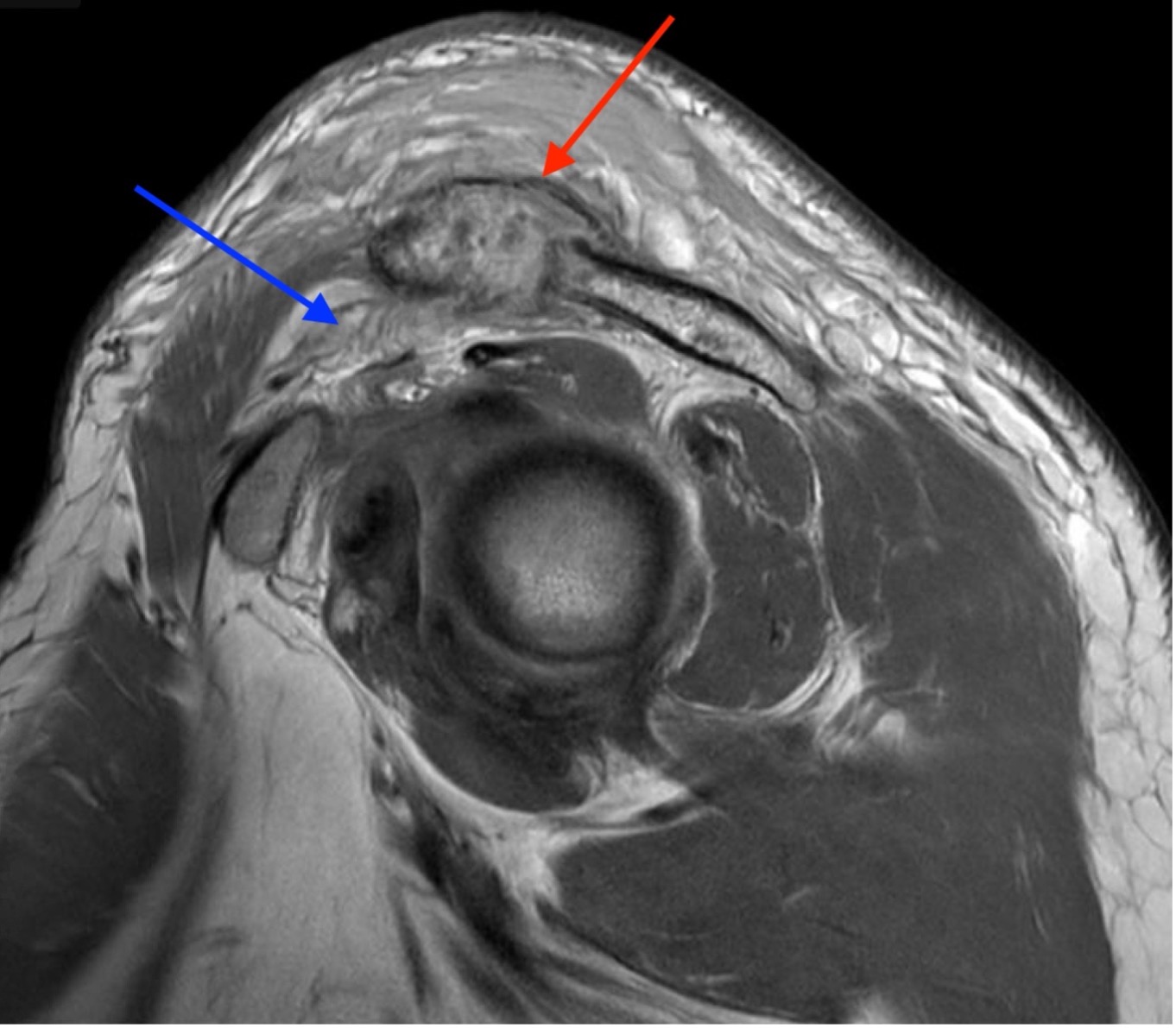

Supplement: Supplementary file 3 [file jetem-9-1-V9-supp3.jpg]

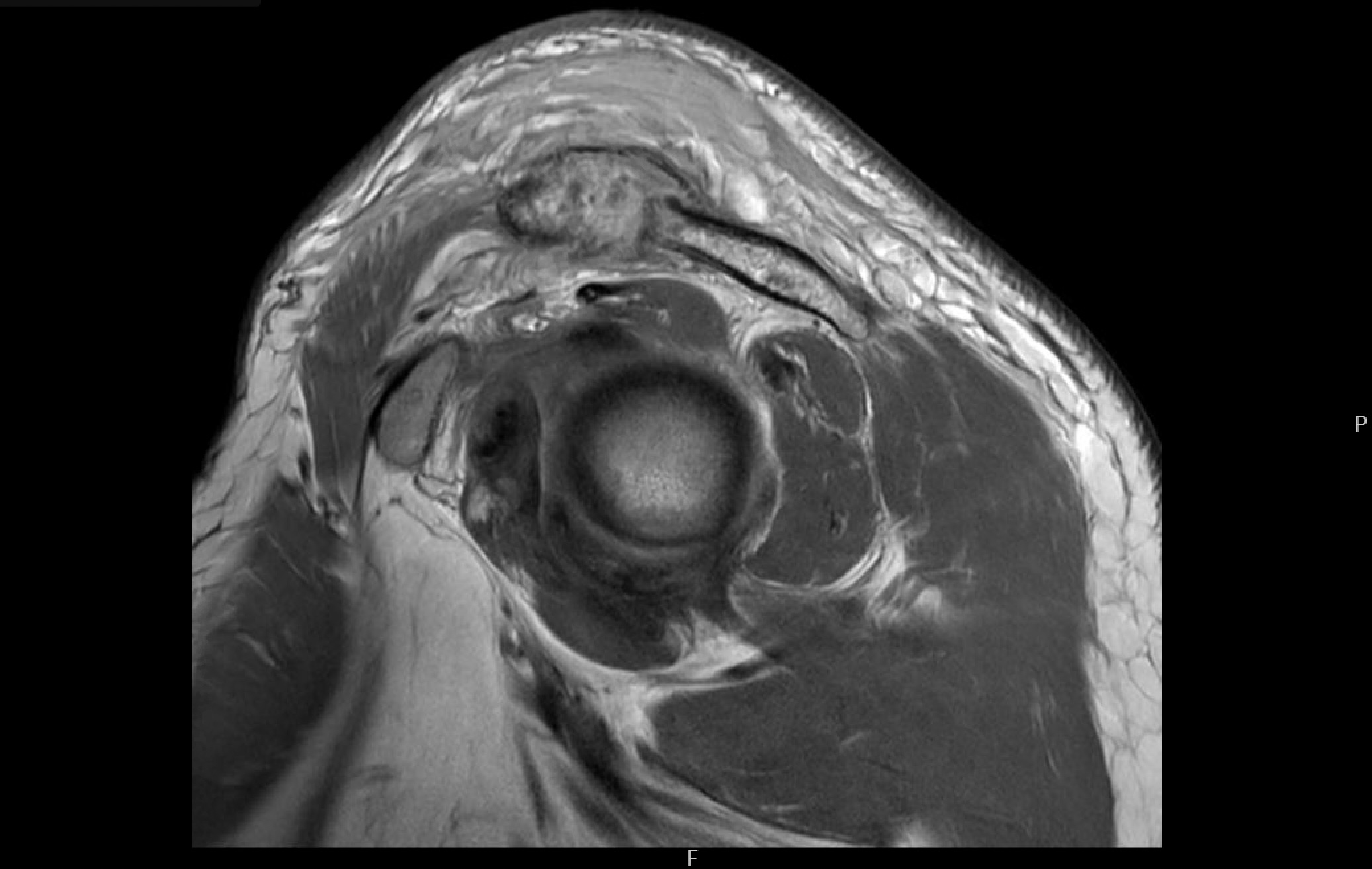

Supplement: Supplementary file 4 [file jetem-9-1-V9-supp4.jpg]

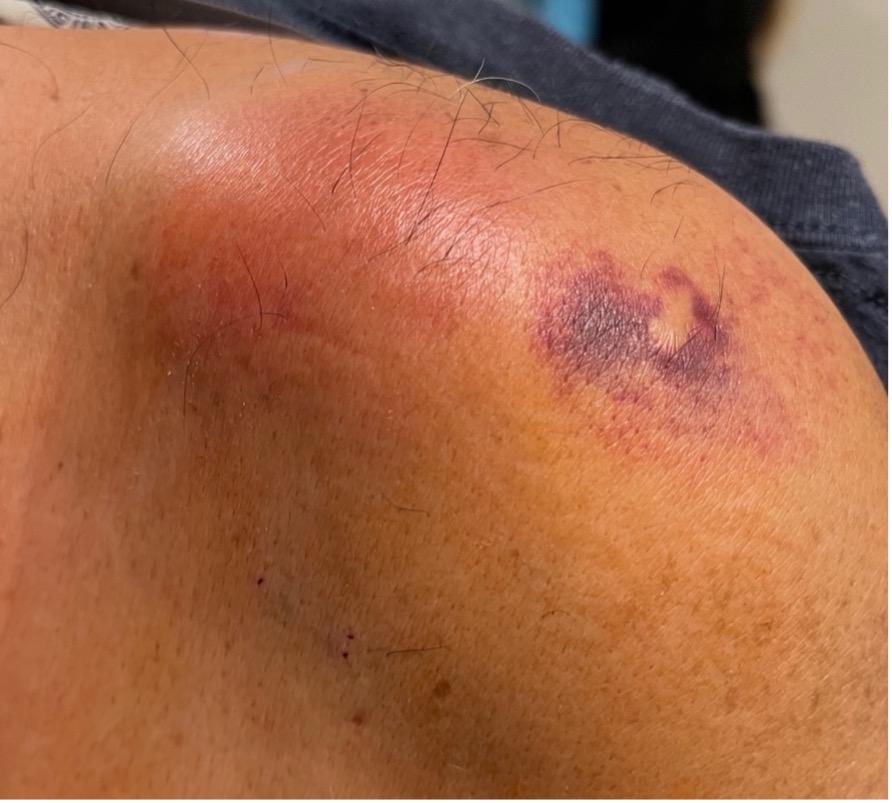

Supplement: Supplementary file 5 [file jetem-9-1-V9-supp5.jpg]

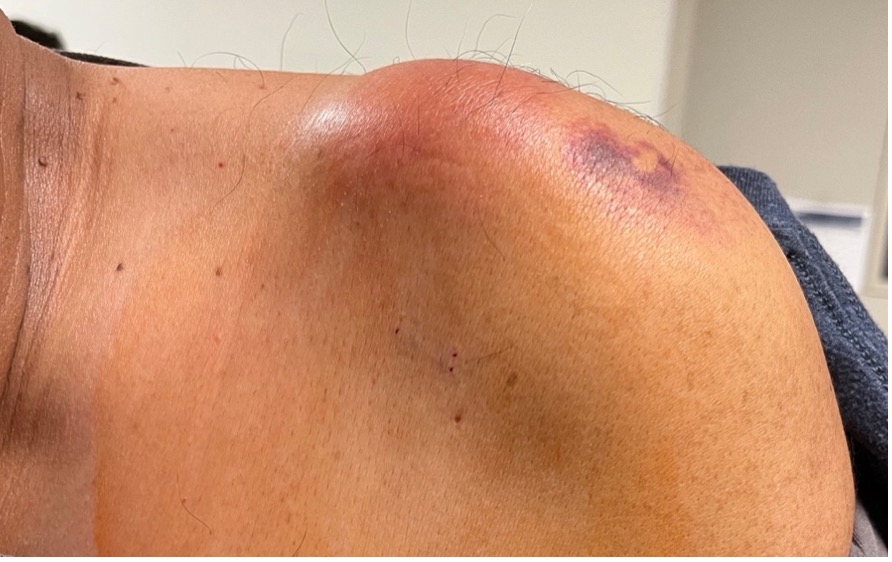

Supplement: Supplementary file 6 [file jetem-9-1-V9-supp6.jpg]

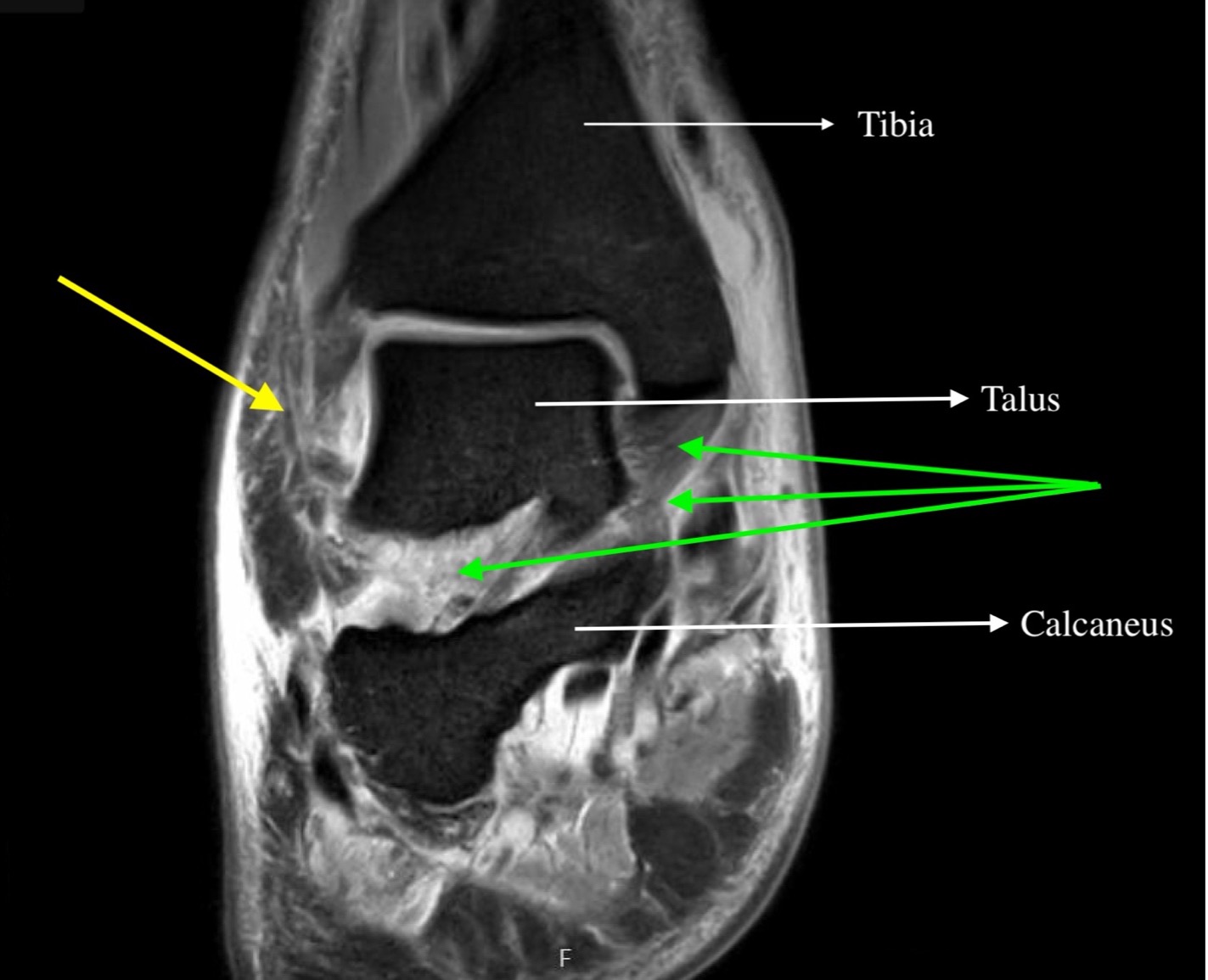

Supplement: Supplementary file 7 [file jetem-9-1-V9-supp7.jpg]

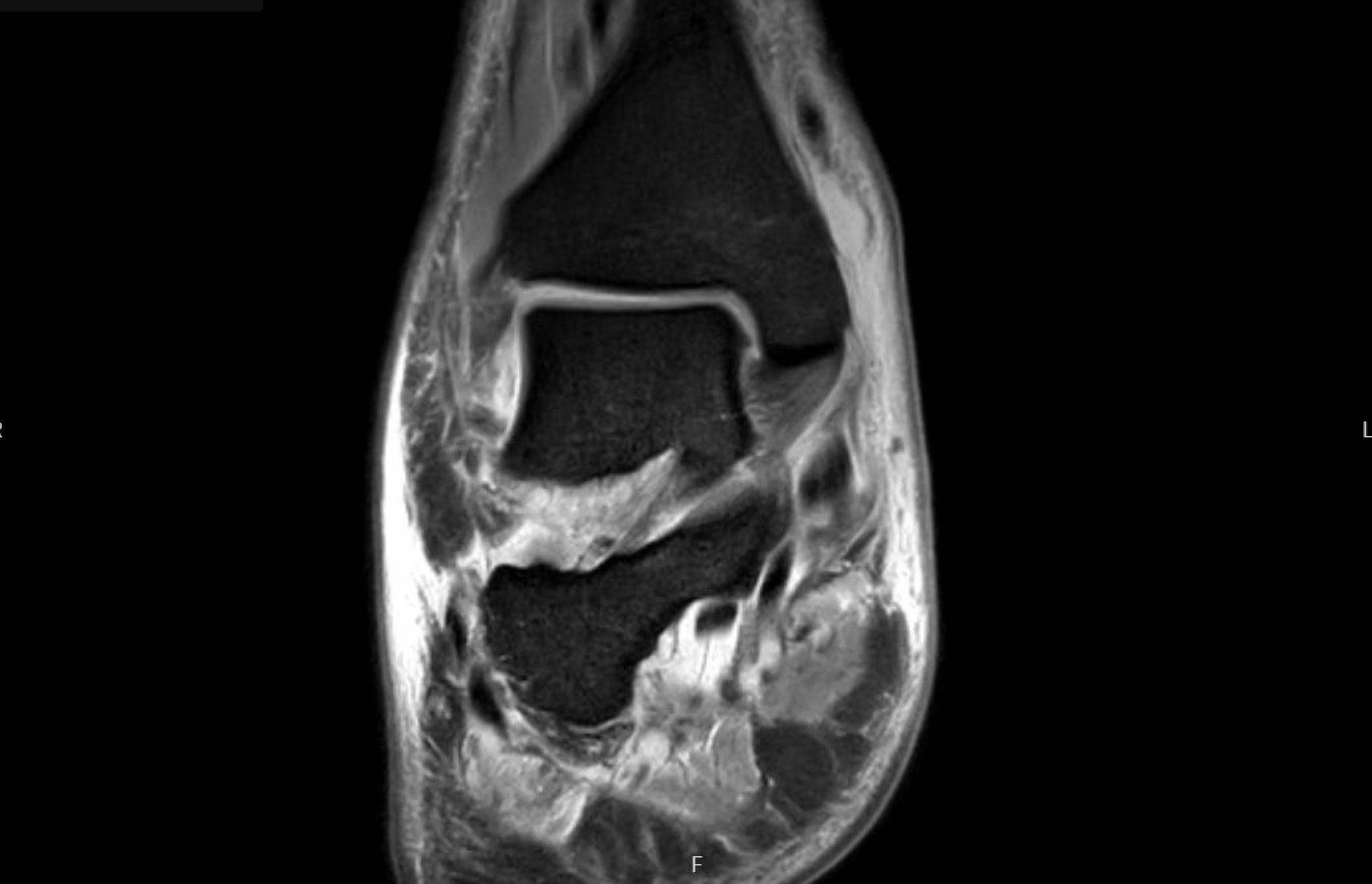

Supplement: Supplementary file 8 [file jetem-9-1-V9-supp8.jpg]
